# Clinical significance of stromal apoptosis in colorectal cancer

**DOI:** 10.1038/sj.bjc.6605220

**Published:** 2009-08-04

**Authors:** P J Koelink, C F M Sier, D W Hommes, C B H W Lamers, H W Verspaget

**Affiliations:** 1 Department of Gastroenterology-Hepatology, Leiden University Medical Center, Leiden, The Netherlands

**Keywords:** stroma, tumour, apoptosis, prognosis, colorectal cancer

## Abstract

**Background:**

Epithelial and stromal cells play an important role in the development of colorectal cancer (CRC). We aimed to determine the prognostic significance of both epithelial and stromal cell apoptosis in CRC.

**Methods:**

Total apoptosis was determined by caspase-3 activity measurements in protein homogenates of CRC specimens and adjacent normal mucosa of 211 CRC patients. Epithelial apoptosis was determined by an ELISA specific for a caspase-3-degraded cytokeratin 18 product, the M30 antigen. Stromal apoptosis was determined from the ratio between total and epithelial apoptosis.

**Results:**

Epithelial and stromal apoptosis, as well as total apoptosis, were significantly higher in CRC compared with corresponding adjacent normal mucosa. Low total tumour apoptosis (⩽median caspase-3 activity) was associated with a significantly worse disease recurrence (hazard ratio (HR), 95% confidence interval (95% CI): 1.77 (1.05–3.01)), independent of clinocopathological parameters. Epithelial apoptosis was not associated with clinical outcome. In contrast, low stromal apoptosis (⩽median caspase-3/M30) was found to be an independent prognostic factor for overall survival, disease-free survival and disease recurrence, with HRs (95% CI) of 1.66 (1.17–2.35), 1.62 (1.15–2.29) and 1.69 (1.01–2.85), respectively.

**Interpretation:**

Stromal apoptosis, in contrast to epithelial apoptosis, is an important factor with respect to survival and disease-recurrence in CRC.

The classical normal mucosa–adenoma–carcinoma sequence of colorectal cancer (CRC) is associated with a resistance to apoptosis (Koornstra *et al*, 2003). The use of apoptosis indices or measurements as a prognostic or predictive factor for CRC has been limited by the reliability of the detection techniques and time consuming evaluations, and have shown conflicting results (de Bruin and Medema, 2008). Caspases, or death proteases, are cysteine proteases that are responsible for the morphological changes within cells during apoptosis (Kumar, 2007). Caspase-3 is at the point of convergence of the two main apoptotic pathways and cleaves most of the cellular substrates in the apoptotic process (Porter and Janicke, 1999). Measurement of caspase-3 activity is therefore a major and the most reliable determinant of apoptosis (Kaufmann *et al*, 2008). Caspase-3-degraded proteins are also used to detect apoptosis, for instance by the M30 antibody that detects an epithelial-specific caspase-3-degraded product of cytokeratin 18 (CK18), that is, CK18-Asp396, and therefore specifically detects apoptotic epithelial cells (Leers *et al*, 1999).

In recent years it has become clear that stromal cells within the tumour, and their interactions with the tumour cells, are important in the initiation and progression of cancer (Mueller and Fusenig, 2004), illustrated by the prognostic relevance of tumour stromal-epithelial ratios for CRC patients (Mesker *et al*, 2007). Apoptosis of stromal cells might therefore also be of prognostic importance for the patients. To investigate the prognostic impact of stromal apoptosis, we determined total cellular apoptosis by caspase-3 activity measurement, and epithelial apoptosis by an ELISA for caspase-3-degraded CK18 (M30 antigen), in a large series of CRC specimens. Correction of the total apoptosis for the epithelial apoptosis (caspase-3/M30) revealed the apoptosis of non-epithelial cells, that is, stromal apoptosis, which was found to be of major clinical relevance in the CRC patients.

## Materials and methods

### Patients

The study population consisted of 211 CRC patients that were admitted to the Leiden University Medical Center for tumour resection between December 1983 and September 1991, as previously described (Langers *et al*, 2008). Fresh tissue was collected from the non-irradiated surgical specimen immediately after resection and attention was paid to collect material from the non-necrotic mid-central part of the tumour. Normal mucosa samples were obtained whenever possible at a distance of approximately 10 cm from the tumour. Tissue samples were frozen and stored at −70°C until use. Clinical data and follow-up information were available for a period of at least 5 years after surgical intervention. Macroscopic (diameter and localisation of the tumour) as well as microscopic data were assessed, including Dukes' stage and differentiation (and ulceration) grade of the tumour according to the WHO classification. Colonic cancers were classified as being proximal or distal to, and including, the splenic flexure. The study was performed according to the guidelines of the Medical Ethics Committee of the Leiden University Medical Center and in compliance with the Helsinki Declaration.

### Tissue homogenisation and protein determination

Frozen tissue specimens were weighed and homogenised on ice for 2 min in 1 ml Tris-HCl, 0.1% Tween 80, pH 7.5 per 60 mg tissue using a Potter device (B Braun, Germany), and centrifuged twice at 8000 **g** for 2.5 min at 4°C, as described before (Langers *et al*, 2008). Protein content was measured according to Lowry *et al.* (1951) and standardised by bovine serum albumin (BSA).

### Caspase-3 activity measurement

For measurement of caspase-3 enzymatic activity, a colorimetric assay was used as described before (de Heer *et al*, 2007). Samples were incubated with a saturating concentration of 25 *μ*M specific enzyme substrate Ac-Aps-Glu-Val-Asp-AMC (Ac-DEVD-AMC; Bachem, Heidelberg, Germany) in a total of 100 *μ*l 100 mM HEPES buffer, pH 7.25, containing 10% (w/v) sucrose, 0.1% (v/v) Nonidet-P40 and 10 mM dithiothreitol. During incubation at 37°C, fluorescent AMC release by active caspase-3 was monitored at an excitation of 360 nm and an emission of 460 nm using a Fluostar Optima plate reader (BMG Labtech GMBH, Offenburg, Germany). Calibration curves were constructed using free AMC. Caspase-3 activity was indicated in pmol AMC min^−1^ mg^−1^ protein. The intra- and interassay variabilities of the caspase-3 activity assay were between 5 and 15%.

### M30 and CK18 antigen level detection

M30 antigen and total CK18 antigen levels were detected by M30 and M65 ELISAs, according to the manufacturer's protocol (Peviva BV, Bromma, Sweden), and expressed as antigen units per mg protein (U mg^−1^). Both ELISAs had intra- and interassay variabilities <10%, in line with the manufacturer's specifications.

### Western blotting for caspase-3

Tumour protein samples were separated on a 12% polyacrylamide SDS gel and blotted to a nitrocellulose membrane (Whatman, Dassel, Germany). After blocking with 2.5% milk powder (Bio-Rad Laboratories, Hercules, CA, USA) in 0.05% Tween 20 in phosphate-buffered saline (PBST) for 2 h at room temperature (RT) blots were subsequently incubated overnight at 4°C with a rabbit-anti-total-caspase-3 antibody (1 : 500; Cell Signaling Technology, Danvers, MA, USA) in 0.5% BSA/PBST and Horseradish-peroxidase (HRP)-labelled mouse-anti-rabbit secondary antibody (1 : 1000; Dako, Glostrup, Denmark) for 2 h at RT. The signal was developed with Supersignal West Pico Chemiluminescent Substrate (Pierce, Rockford, IL, USA), and detected by an LCD camera (Bio-Rad).

### Immunohistochemistry for active caspase-3, M30 antigen and cytokeratin

Paraffin tissue sections (5 *μ*m) were deparaffinised, blocked in 0.3% H_2_O_2_ in methanol for 30 min at RT and rehydrated through graded ethanol to phosphate-buffered saline (PBS). Antigen retrieval was performed by boiling the slides in 10 mM citrate buffer (pH 6.0) for 10 min in a microwave oven. After cooling down to RT and rinsing with PBS for three times the slides were blocked with 5% goat serum (Dako) in 1% BSA in PBS for 20 min at RT. Afterwards the sections were incubated with the primary antibody in 1% BSA/PBS overnight at 4°C: mouse-anti-pan-cytokeratin (1 : 5000, clone C11; Santa Cruz Biotechnologies, Santa Cruz, CA, USA), mouse M30 Cytodeath antibody (1 : 400; Roche Applied Science, Penzberg, Germany) and rabbit-anti-active-caspase-3 (1 : 200; Cell Signaling Technology). After washing with PBS, the slides were incubated with biotinylated goat-anti-mouse or goat-anti-rabbit secondary antibodies (both 1 : 200; Dako) in 1% BSA/PBS for 45 min. After thorough washing with PBS, the slides were incubated with streptavidin–avidin–biotin complex/HRP (Dako) for 45 min at RT. The signal was developed with 0.015% H_2_O_2_ (Merck, Darmstadt, Germany) in 0.05% diaminobenzidine-tetrahydrochloride (Sigma, Zwijndrecht, The Netherlands) in 0.01 M Tris-HCL pH 7.6 for 10 min resulting in a brown staining product. Sections were counterstained with Mayers' haematoxylin (Merck), dehydrated and mounted in entallan (Merck). Slides without primary antibody incubations were included as negative controls. Photomicrographs of representative sections were taken with a Nikon Elipse E800 microscope equipped with a Nikon DXM 200 camera.

### Statistical analysis

Statistical analysis was performed with Statistical Package for Social Sciences (SPSS) statistical software (version 12.0 for Windows; SPSS Inc., Chicago, IL, USA). Wilcoxon signed rank and Spearman's rho tests were used to compare paired observations that did not follow a normal distribution. Kruskall–Wallis and Mann–Whitney *U*-tests were used to compare two or more groups that do not follow a normal distribution. The entry data for survival analysis were the time of surgery for the primary tumour. Biomarker expressions were dichotomised and overall survival, disease recurrence and disease-free survival were evaluated using Kaplan–Meier methodology with death, local and distant recurrence, or both, as events, including log-rank tests. Univariate Cox proportional hazard models were used to explore the association of markers with clinical outcome. Variables with a *P*⩽0.1 in the univariate analyses were subjected to multivariate analyses. Statistical tests were two-sided and *P*<0.05 was considered statistically significant. Graphs were made with Graphpad Prism (version 4.0; Graphpad Prism Inc., La Jolla, CA, USA) software.

## Results

### Total, epithelial and stromal apoptosis in colorectal cancer

The caspase-3 activity, M30 antigen and CK18 levels were determined in colorectal carcinoma tissue of all 211 patients, whereas due to tissue/homogenate availability these parameters could only be determined in adjacent normal tissue from 177 patients. M30 antigen, CK18 and caspase-3 activity levels in the colorectal tumour tissue samples were significantly higher than those in the corresponding normal tissue samples in these 177 patients (Table 1).

**Figure 1:**
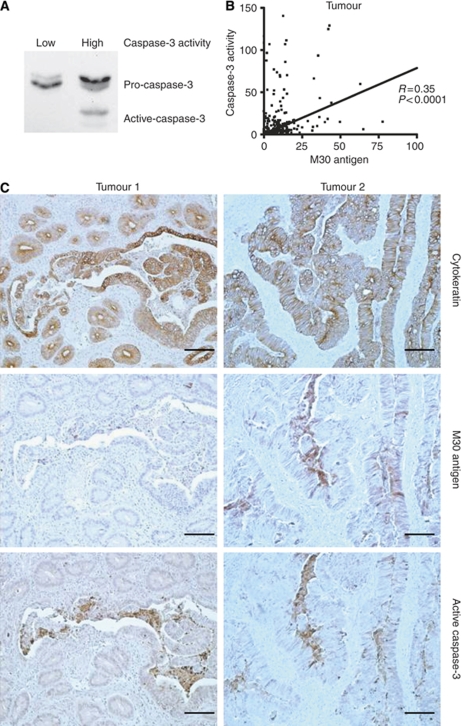
Caspase-3 activity in CRC. CRC specimens with high caspase-3 activity show levels of the active form of caspase-3, whereas low caspase-3 activity CRC specimens only show inactive (pro-) caspase-3 as determined by immunoblotting for total caspase-3 (**A**). Caspase-3 activity and M30 antigen in CRC show a weak but significant correlation (**B**). Some tumours show high caspase-3 activity without high levels of M30 antigen, suggesting stromal apoptosis. Photomicrographs of immunohistochemical stainings for active caspase-3, M30 and pan-cytokeratin showing tumours with stromal cells expressing active caspase-3 (**C**), as represented by Tumour 1 (left panel), and tumours with mainly epithelial cells expressing active caspase-3, correlating with M30 staining (represented by Tumour 2, right panel). Scale bars=100 *μ*m.

Immunoblot analysis confirmed that tumours with a high caspase-3 activity had high protein levels of active caspase-3 compared with low caspase-3 activity tumours (Figure 1A). Overall there was a weak but significant correlation between tumour caspase-3 activity and tumour M30 antigen levels, as expected, because the M30 antigen is generated by active caspase-3 (Figure 1B). Some tumours had really high caspase-3 activity levels, without high M30 antigen levels, indicating high stromal apoptosis. Immunohistochemical analysis confirmed that in these tumours active caspase-3 was indeed mainly expressed by diverse non-epithelial cells and were almost M30 antigen-negative (Figure 1C, represented by Tumour 1), compared with other tumours in which epithelial cells were expressing most of the active caspase-3 and M30 antigen-positive (represented by tumour 2). Some high power magnifications of tumour 1 to illustrate the diverse nature of stromal apoptosis are shown in Supplementary Figure 1. Our measurements enabled us to further differentiate in cell type origin of apoptosis by calculating the percentage of apoptotic epithelial cells through the formula M30/CK18 × 100 and for stromal apoptosis through the formula caspase-3/M30. This epithelial as well as stromal apoptosis was also significantly higher in tumour tissue compared with corresponding normal tissue (Table 1).

### Relation between total, epithelial and stromal apoptosis and clinical variables

The association between the clinicopathological characteristics of the 211 colorectal cancer patients and the tissue caspase-3 activity, as a measure of total apoptosis, is summarised in Table 2. Caspase-3 activity level in the tumours correlated with gender, tumour location and Dukes' stage, that is, significantly higher in females (*P*=0.006) and in tumours located in the proximal colon (*P*=0.02), and decreased with increasing Dukes' stage (*P*=0.01). Caspase-3 activity in normal mucosa was also found to be higher in females (*P*=0.04) and in the proximal colon (*P*=0.04), but did not correlate with Dukes' stage.

Tumour M30 antigen levels correlated with grade of differentiation, with well-differentiated tumours having significantly higher levels (Table 3; *P*=0.02). The total amount of epithelial cells (CK18 per mg protein) in the tumour samples correlated with the tumour diameter, larger tumours having significantly lower amounts of epithelial cells compared with smaller tumours (*P*=0.007). Epithelial cell apoptosis (M30/CK18 × 100) was significantly higher in tumours with ulceration (*P*=0.02). Stromal apoptosis (caspase-3/M30 ratio) was related to gender and tumour location, that is, significantly higher in females (*P*=0.02) and proximal colon tumours (*P*<0.001).

### Stromal apoptosis predicts overall survival, disease-free survival and disease recurrence

**Figure 2:**
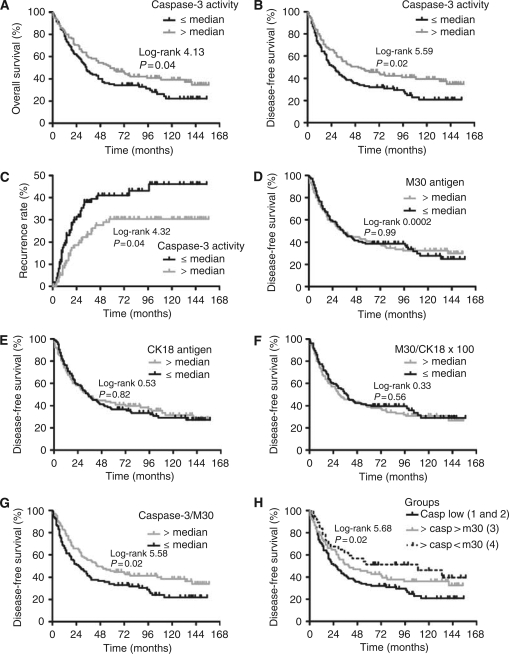
Clinical outcome of CRC patients in relation to caspase-3 activity, M30 and CK18 antigen levels. Kaplan–Meier overall survival (**A**), disease-free survival (**B**, **D**–**H**) and recurrence (**C**) curves of all CRC patients (*n*=211), groups divided on median tumour caspase-3 activity (A–C), median M30 antigen (D), median CK18 (E), median M30/CK18 × 100 (F), median caspase-3/M30 (G) and both median caspase-3 and median M30 levels (H). Log-rank test were used to compare the curves.

High caspase-3 activity (>median) in the tumour was associated with better overall patient survival; median overall survival: 58 *vs* 34 months (Figure 2A). These patients with high tumour caspase-3 activity (>median) also had a significantly better disease-free survival with a median disease-free survival of 47.5 *vs* 27 months for patients with low tumour caspase-3 activity (⩽median; Figure 2B). Low tumour caspase-3 activity (⩽median) was also accompanied by a shorter time to recurrence compared to patients with a high tumour caspase-3 activity (Figure 2C), with 5-year recurrence rates of 46.1% and 30.3%, respectively. Caspase-3 activity in the normal adjacent mucosa was not associated with overall survival, disease-free survival or disease recurrence of the patients (not shown).

Tumour M30 antigen, CK18 or M30/CK18 × 100 levels did not show any relation with disease-free survival (Figure 2D, E and F), overall survival or disease recurrence (not shown). In contrast, the caspase-3/M30 ratio of the tumours correlated very strongly with the disease-free survival of the patients (Figure 2G), with a high ratio (>median) having a significantly better median disease-free survival of 45 *vs* 26.5 months for the patients with tumours with a low ratio.

To underline the importance of stromal apoptosis to the clinical outcome of the CRC patients, we divided our patient population in four groups based on median tumour M30 antigen and median tumour caspase-3 activity levels: (1) low caspase-3/M30 low (*n*=67), (2) low caspase-3/M30 high (*n*=39), (3) caspase-3 high/M30 high (*n*=68) and (4) caspase-3 high/M30 low (*n*=37). The M30 antigen levels did not have an additive effect in the two groups with low caspase-3 activity, and therefore groups 1 and 2 were pooled. In the groups with high caspase-3 activity, the disease-free survival was found to be the best for patients with a high tumour caspase-3 activity and low M30 antigen level (Figure 2H; group 4 *vs* groups 1 and 2, log-rank value: 5.68, *P*=0.02), which strengthened our notion that stromal apoptosis in the tumour is an important prognostic factor for the patients' disease-free survival.

The estimates of relative risk of the evaluated parameters to disease outcome, as calculated by hazard ratios in the univariate Cox regression analysis, are shown in Table 4. The clinicopathological factors that were significantly associated with worse outcome were found to be old age and advanced Dukes' stage, as expected. With regard to the apoptosis parameters, a low total (caspase-3 activity) or low stromal (caspase-3/M30) apoptosis were consistently found to be associated with a worse overall and disease-free survival as well as with a higher risk of recurrence. Adjustment of the apoptosis parameters to the most relevant clinicopathological parameters, that is, gender, age, Dukes' stage, tumour localisation and tumour diameter in the multivariate Cox proportional hazards analysis, identified stromal apoptosis as an independent prognostic factor for overall survival, disease-free survival and disease recurrence. In contrast, total apoptosis was found to be an independent prognostic factor for disease recurrence only (Table 4).

## Discussion

This study shows that stromal apoptosis in CRC is a major determinant for the clinical outcome of patients. The stromal compartment of colorectal carcinomas is populated by a wide variety of cell types, such as immune cells, fibroblasts, myofibroblasts and endothelial cells. Recent studies have demonstrated that genetic instability of both the stromal and epithelial compartment contributes to the genesis of CRC (Ishiguro *et al*, 2006). Important signalling pathways that are frequently disrupted in CRC, such as the transforming growth factor (TGF)-*β* (Naber *et al*, 2008), bone morphogenic protein (BMP; Hardwick *et al*, 2008) and Wnt pathway (Macheda and Stacker, 2008), play a role in both the cancer-associated stromal and epithelial compartment in the development of CRC. The presence of fibroblasts (Ngan *et al*, 2007), myofibroblasts (Tsujino *et al*, 2007) and endothelial cells (Baeten *et al*, 2006) in the cancer-associated stroma contributes to progression of CRC and a worse patient prognosis. Also, the expression of other proteins in the cancer-associated stroma, that is, hypoxia regulated proteins (Cleven *et al*, 2007), matrix degrading proteases (Hilska *et al*, 2007) and cyclooxygenase-2 (Sonoshita *et al*, 2002; Adegboyega *et al*, 2004), is correlated to CRC progression and disease prognosis. The importance of the cancer-associated stroma in CRC is most simply illustrated by the reported prognostic significance of the tumour-stroma ratio in CRC (Mesker *et al*, 2007). The cancer-associated stromal cells, and their interaction with the epithelial tumour cells, are therefore speculated to be important targets for future therapy (Mueller and Fusenig, 2004).

Programmed cell death, that is, apoptosis, is an important mechanism to maintain tissue homoeostasis. Defects in the two main apoptotic pathways, the extrinsic and intrinsic pathways, lead to a resistance to apoptosis and increased cell survival. Epithelial apoptosis in CRC has been widely studied but still the significance for the prognosis of CRC patients, as well as the significance for the response to treatment such as chemotherapy, is unclear (Koornstra *et al*, 2003; de Bruin and Medema, 2008). As apoptosis in the cancer-associated stroma could also play an important role, and its induction could be relevant for treatment response, we investigated the contribution of stromal apoptosis, as well as epithelial apoptosis in the clinical outcome of CRC.

We showed that both epithelial and stromal apoptosis, as well as total apoptosis, were significantly higher in non-irradiated CRC than in normal adjacent mucosa, as found in other studies (Leonardos *et al*, 1999; Jonges *et al*, 2001; Koornstra *et al*, 2003; de Heer *et al*, 2007; de Oca *et al*, 2008). As cancer cells usually have apoptotic defects and are more difficult to drive into apoptosis (Igney and Krammer, 2002; Brown and Attardi, 2005), higher levels of apoptosis in cancer, compared with normal tissue, might seem to be somewhat unexpected. However, the level of apoptosis and sensitivity to apoptotic signals are two, although related, different things. Importantly, in this study, total CRC apoptosis decreased with increasing Dukes' stage, whereas no such correlation was found in adjacent normal mucosa, which indeed indicates that cells in the tumour become resistant to apoptotic signals during tumour progression resulting in a decreased caspase-3 activity in more advanced tumour stages. Moreover, total apoptosis in the tumour showed a strong association with risk of recurrence and disease-free survival. Cox multivariate hazards analysis identified low caspase-3 activity in the tumour as an independent risk factor of recurrence, with patients with tumour caspase-3 activity below the median value having a 1.77 times higher relative risk of recurrence, similar as reported by others for both colon and rectal cancer (de Heer *et al*, 2007; de Oca *et al*, 2008). Caspase-3 activity corrected for the percentage of epithelial cells, as determined on slides, showed similar results compared with the uncorrected caspase-3 activity in rectal cancer in the study by de Heer *et al* (2007), and therefore the authors concluded that caspase-3 activity is a prognostic factor for local recurrence in rectal cancer without the previous knowledge of epithelial-stromal ratios in the tumour. Correction of caspase-3 activity for the amount of epithelial cells (measured by CK18 antigen levels), caspase-3/CK18, in this study indeed showed similar results with respect to clinicopathological parameters and clinical outcome, as caspase-3 activity alone (not shown). However, this does not indicate the source of the caspase-3 activity; it could be that most of the caspase-3 activity is produced by apoptotic epithelial cells, and the apoptotic stromal cells could also be an important source. To investigate the source we determined M30 antigen levels by ELISA in the same CRC homogenates, as a measurement of epithelial caspase-3 activity/apoptosis (Leers *et al*, 1999; Hagg *et al*, 2002). If apoptotic epithelial cells were mainly responsible for the caspase-3 activity in this study, M30 antigen levels should have shown a similar correlation with patient and tumour characteristics, which was clearly not the case. Our measurement of epithelial apoptosis, determined by M30/CK18 × 100 (%), with median values of 3.72% for tumour tissue and 1.83% for normal tissue, is very much in line with epithelial apoptosis found in other studies (Koornstra *et al*, 2003). M30 antigen levels, either corrected for CK18 or uncorrected, did not correlate with overall survival, disease-free survival or disease recurrence, in contrast to caspase-3 activity, indicating the importance of non-epithelial caspase-3 activity, that is, stromal apoptosis. This was confirmed by determining caspase-3/M30 ratios, as stromal apoptosis levels, which even showed a stronger association with overall survival, disease-free survival or disease recurrence. Low stromal apoptosis (⩽median caspase-3/M30) was an independent prognostic factor for overall survival, disease-free survival and disease recurrence with an HR (95% CI) of 1.66 (1.17–2.35), 1.62 (1.15–2.29) and 1.69 (1.01–2.85), respectively.

Epithelial tumour cell apoptosis has been extensively studied in CRC, but still the relevance of the apoptotic index as a prognostic factor is a matter of debate (Koornstra *et al*, 2003; de Bruin and Medema, 2008). The detection technique is likely to influence the apoptotic levels, as some detection techniques are really specific for epithelial apoptotic cells, that is, M30 immunohistochemistry and M30 ELISA (Leers *et al*, 1999; Hagg *et al*, 2002), and others are (more or less) specific for apoptotic cells in general, that is, TdT-mediated dUTP-biotin nick end-labeling (TUNEL) (Stadelmann and Lassmann, 2000; Barrett *et al*, 2001), active caspase-3 immunohistochemistry or caspase-3 activity measurements (Kaufmann *et al*, 2008), and also detect apoptotic stromal cells. The results presented here, that is, stromal apoptosis as an important prognostic factor for CRC, suggests that discrepancies found in the literature might be explained by the different detection techniques that are used, that is, ‘specific for epithelial apoptosis’ *vs* ‘specific for apoptosis in general’.

In conclusion, this study confirms that caspase-3 activity within tumour tissue is an important denominator of disease recurrence and patient survival in CRC, with high levels of caspase-3 associated with good outcome. Remarkably, this caspase-3 activity was found to predominantly reside within the non-epithelial compartment of the tumours. These observations underline the importance of stromal cell apoptosis in CRC progression and identify the cancer-associated stroma as a potential therapeutic target.

## Figures and Tables

**Table 1:** Caspase-3 activity, M30 antigen and CK18 levels in tumour tissue and normal adjacent mucosa pairs from 177 colorectal cancer patients

**Tissue assessed/parameter**	**Tumour median (IQR)**	**Normal mucosa median (IQR)**	***P* value**
Caspase-3 activity (pmol AMC mg^−1^ min^−1^)	6.74 (2.96-16.65)	1.28 (0.66-2.20)	<0.0001
M30 antigen (U mg^−1^)	7.58 (3.13-13.77)	3.20 (1.13-5.66)	<0.0001
CK18 (U mg^−1^)	209.6 (116.9-273.1)	178.5 (129.6-132.5)	0.008
M30/CK18 × 100 (epithelial apoptosis)	3.72 (2.52-5.78)	1.83 (0.86-3.16)	<0.0001
Caspase-3/M30 (stromal apoptosis)	0.84 (0.44-2.97)	0.38 (0.18-1.18)	<0.0001

Epithelial apoptosis (M30/CK18 × 100) and stromal apoptosis (caspase-3/M30) were calculated from caspase-3, M30 antigen and CK18 levels. *P* values were determined with Mann–Whitney *U*-test.

IQR=interquartile range.

**Table 2:** Caspase-3 activity in relation to patient and tumour characteristics

	**Tumour**			**Normal mucosa**		
**Tissue Patient and tumour characteristics**	**No. of patients (%); total *n*=211**	**Caspase-3 activity median (IQR)**	***P* value**	**No. of patients (%); total *n*=177**	**Caspase-3 activity median (IQR)**	***P* value**
*Gender*			**0.006**			**0.04**
Male	118 (56)	5.93 (1.96–14.55)		102 (58)	1.07 (0.59–2.19)	
Female	93 (44)	9.77 (3.89–26.00)		75 (42)	1.40 (0.78–2.21)	
						
*Age (median 69 years; range 31–90)*			0.48			0.60
<Median	105 (50)	7.33 (2.76–16.10)		88 (50)	1.23 (0.75–2.05)	
⩾Median	106 (50)	7.10 (3.39–19.34)		89 (50)	1.37 (0.62–2.38)	
						
*Location*			**0.02**			**0.04**
Right or transverse	73 (35)	10.79 (3.43–47.72)		62 (35)	1.40 (0.80–2.67)	
Left colon or sigmoid	72 (34)	5.75 (1.83–16.76)		59 (33)	1.37 (0.70–2.10)	
Rectum	66 (31)	5.25 (2.94–14.47)		56 (32)	0.99 (0.52–1.93)	
						
*Dukes' stage*			**0.01**			0.66
A/B	122 (58)	9.00 (3.51–20.35)		106 (60)	1.25 (0.66–2.21)	
C	57 (27)	6.19 (2.36–15.27)		45 (25)	1.18 (0.61–2.08)	
D	32 (15)	3.80 (1.24–13.16)		26 (15)	1.38 (0.70–2.34)	
						
*Tumour ∅ (median 5 cm; range 2–12)*			0.47			0.98
<Median	93 (44)	5.98 (3.37–15.64)		79 (45)	1.31 (0.72–2.00)	
⩾Median	118 (56)	8.38 (2.89–19.36)		98 (55)	1.21 (0.63–2.27)	
						
*Grade of differentiation* a			0.10			0.36
Poor	25 (12)	8.23 (3.76–14.77)		21 (12)	2.00 (0.75–2.97)	
Moderate	143 (68)	13.46 (4.02–27.33)		122 (69)	1.25 (0.64–2.14)	
Well	40 (19)	7.01 (3.79–40.51)		31 (18)	1.28 (0.59–2.19)	
						
*WHO classification* a			0.29			0.31
Adenocarcinoma	157 (75)	7.65 (3.36–17.60)		137 (77)	1.23 (0.64–2.09)	
Mucinous carcinoma	52 (25)	6.03 (1.75–15.92)		39 (22)	1.39 (0.72–2.62)	
						
*Ulceration* a			0.82			0.59
No	48 (27)	6.73 (3.23–25.65)		44 (25)	1.25 (0.74–2.26)	
Yes	139 (73)	7.33 (2.94–16.59)		118 (66)	1.30 (0.62–2.17)	

*P* values were calculated with Kruskall–Wallis and Mann–Whitney *U*-tests, and *P* values⩽0.05 were considered statistically significant, shown in bold.

IQR=interquartile range.

a Some values were missing.

**Table 3:** Tumour M30 antigen, CK18, M30/CK18 x 100 and caspase-3/M30 ratio in relation to patient and tumour characteristics of colorectal cancer patients

**Parameter/patient and tumour characteristics**	**Tumour M30 (U mg^−1^); median (IQR)**	***P* value**	**Tumour CK18 (U mg^−1^); median (IQR)**	***P* value**	**Tumour M30/CK18 × 100; median (IQR)**	***P* value**	**Tumour caspase-3/M30; median (IQR)**	***P* value**
*Gender*		0.34		0.72		0.53		**0.02**
Male	8.7 (3.6–15.2)		218.7 (128.6–287.9)		3.9 (2.5–6.7)		0.8 (0.3–2.2)	
Female	8.1 (3.2–13.7)		204.8 (124.4–291.8)		3.8 (2.3–5.4)		1.0 (0.5–4.9)	
								
*Age*		0.94		0.38		0.22		0.43
<Median	8.7 (3.1–15.2)		224.5 (127.6–296.7)		3.6 (2.2–5.9)		0.8 (0.4–4.0)	
⩾Median	8.4 (3.9–13.8)		214.6 (125–7–263.7)		4.0 (2.9–5.8)		0.9 (0.4–2.9)	
								
*Location*		0.06		0.17		0.24		**<0.001**
Right or transverse	6.8 (2.4–12.7)		189.0 (124.8–257.8)		3.6 (1.8–6.3)		2.1 (0.6–7.6)	
Left colon/sigmoid	9.2 (3.5–15.5)		208.6 (113.4–288.8)		3.8 (2.7–5.8)		0.8 (0.4–2.2)	
Rectum	9.4 (5.9–15.3)		224.6 (164.9–302.4)		3.9 (3.0–5.7)		0.7 (0.3–1.1)	
								
*Dukes' stage*		0.54		0.93		0.38		0.21
A/B	8.6 (3.5–15.2)		215.3 (128.7–292.5)		3.8 (2.7–6.6)		0.9 (0.5–3.2)	
C	8.8 (3.4–14.9)		224.5 (137.9–278.6)		4.0 (2.5–5.7)		0.8 (0.3–2.8)	
D	6.8 (2.8–12.6)		206.2 (104.0–291.2)		3.5 (2.1–5.0)		0.5 (0.3–2.3)	
								
*Tumour diameter*		0.14		**0.007**		0.42		0.15
<Median	9.6 (3.7–15.2)		234.8 (153.6–316.0)		3.7 (2.3–5.8)		0.7 (0.4–2.4)	
⩾Median	7.2 (3.2–13.7)		192.0 (116.0–262.0)		3.8 (2.7–6.9)		0.9 (0.4–3.3)	
								
*Grade of differentiation*		**0.02**		0.10		0.17		0.35
Poor	7.4 (1.6–15.4)		197.1 (78.1–274.8)		4.9 (2.6–7.0)		1.4 (0.4–5.0)	
Moderate	8.0 (3.2–13.8)		215.1 (122.9–275.1)		3.6 (2.3–5.4)		0.9 (0.4–3.0)	
Well	11.8(5.6–19.8)		226.7 (150.0–400.0)		4.0 (2.9–8.6)		0.8 (0.4–1.8)	
								
*WHO classification*		0.35		0.74		0.29		0.79
Adenocarcinoma	8.7 (3.6–15.0)		214.5 (130.7–291.4)		3.8 (2.7–6.4)		0.8 (0.4–2.9)	
Mucinous carcinoma	7.7 (2.8–14.2)		223.6 (116.6–262.6)		3.7 (2.1–5.4)		0.9 (0.3–3.1)	
								
*Ulceration*		0.36		0.85		**0.02**		0.07
No	5.5 (2.6–15.2)		210.4 (126.0–322.7)		3.1 (2.0–5.1)		1.3 (0.6–4.2)	
Yes	8.6 (3.5–14.1)		217.4 (129.2–280.0)		3.8 (2.8–5.8)		0.8 (0.4–2.9)	

*P* values were calculated with Kruskall–Wallis and Mann–Whitney *U*-tests, and *P*⩽0.05 were considered statistically significant, shown in bold.

IQR=interquartile range.

**Table 4:** Univariate and multivariate Cox regression hazard analysis for overall and disease-free survival, and risk of recurrence including the clinicopathological factors of all patients (*n*=211) and the specified apoptosis assessments

**Outcome/patient and tumour characteristics**	**Overall survival HR (95 % CI)**	***P* value**	**Disease-free survival; HR (95 % CI)**	***P* value**	**Risk of recurrence; HR (95 % CI)**	***P* value**
*Univariate analysis*						
*Gender*						
Female	1 (ref)	0.08	1 (ref)	**0.03**	1 (ref)	0.16
Male	1.35 (0.96–1.89)		1.44 (1.03–2.02)		1.43 (0.87–2.36)	
						
*Age (median 69 years, range 31–90)*						
<Median	1 (ref)	**<0.001**	1 (ref)	**0.001**	1 (ref)	0.26
⩾Median	1.83 (1.31–2.58)		1.78 (1.27–2.49)		1.33 (0.81–2.17)	
						
*Location*						
Right or transverse	1 (ref)		1 (ref)		1 (ref)	
Left colon or sigmoid	1.07 (0.72–1.57)	0.75	1.12 (0.76–1.64)	0.58	1.13 (0.65–1.97)	0.66
Rectum	0.69 (0.45–1.05)	0.08	0.70 (0.46–1.06)	0.09	0.80 (0.40–1.58)	0.52
						
*Dukes' stage*						
A/B	1 (ref)		1 (ref)		1 (ref)	
C	1.93 (1.31–2.84)	**0.001**	2.00 (1.36-2.93)	**< 0.001**	3.35 (2.04–5.52)	**< 0.001**
D	7.54 (4.69–12.12)	**< 0.001**	5.53 (3.50–8.73)	**< 0.001**	0.31 (0.04–2.28)	0.25
						
*Tumour ∅ (median 5 cm, range 2–12)*						
<Median	1 (ref)	0.14	1 (ref)	0.09	1 (ref)	0.12
⩾Median	1.29 (0.92–1.81)		1.34 (0.96–1.87)		1.50 (0.90–2.50)	
						
*Grade of differentiation*						
Poor	1 (ref)	0.16	1 (ref)	0.16	1 (ref)	0.23
Moderate/well	1.42 (0.87–2.34)		1.30 (0.90–1.89)		0.62 (0.28–1.36)	
						
*WHO classification*						
Adenocarcinoma	1 (ref)	0.94	1 (ref)	0.93	1 (ref)	0.73
Mucinous carcinoma	0.99 (0.67–1.45)		0.98 (0.67–1.44)		0.90 (0.51–1.61)	
						
*Ulceration*						
No	1 (ref)	0.26	1 (ref)	0.45	1 (ref)	0.12
Yes	1.28 (0.83–1.98)		1.18 (0.77–1.80)		1.57 (0.82–3.02)	
						
*Tumour caspase-3 activity*						
>Median	1 (ref)	**0.04**	1 (ref)	**0.02**	1 (ref)	**0.04**
⩽Median	1.41 (1.01–1.97)		1.48 (1.07–2.06)		1.68 (1.02–2.78)	
						
*Tumour M30 level*						
>Median	1 (ref)	0.71	1 (ref)	0.90	1 (ref)	0.38
⩽Median	0.94 (0.67–1.31)		0.94 (0.71–1.36)		0.80 (0.49–1.31)	
						
*Tumour CK18 level*						
>Median	1 (ref)	0.99	1 (ref)	0.82	1 (ref)	0.67
⩽Median	1.00 (0.72–1.39)		1.04 (0.75–1.44)		0.90 (0.55–1.47)	
						
*Tumour M30/CK18 × 100 level*						
>Median	1 (ref)	0.52	1 (ref)	0.57	1 (ref)	0.83
⩽Median	0.90 (0.64–1.25)		0.91 (0.65–1.26)		0.95 (0.58–1.55)	
						
*Tumour caspase-3/M30 level*						
>Median	1 (ref)	**0.02**	1 (ref)	**0.02**	1 (ref)	**0.05**
⩽Median	1.51(1.08–2.11)		1.48 (1.06–2.06)		1.64 (1.00–2.70)	
						
*Multivariate analysis*						
Low tumour caspase-3 activity	1.25 (0.87–1.80)	0.22	1.32 (0.92–1.88)	0.13	1.77 (1.05–3.01)	**0.03**
Low tumour caspase-3/M30 level	1.66 (1.17–2.35)	**0.004**	1.62 (1.15–2.29)	**0.006**	1.69 (1.01–2.85)	**0.04**

In the multivariate analyses the apoptosis parameters were adjusted for gender, age, Dukes' stage, tumour diameter and tumour location. *P*⩽0.05 were considered statistically significant, shown in bold.

CI=confidence interval.

## References

[bib1] Adegboyega PA, Ololade O, Saada J, Mifflin R, Di Mari JF, Powell DW (2004) Subepithelial myofibroblasts express cyclooxygenase-2 in colorectal tubular adenomas. Clin Cancer Res 10: 5870–5879 15355919 10.1158/1078-0432.CCR-0431-03

[bib2] Baeten CI, Castermans K, Hillen HF, Griffioen AW (2006) Proliferating endothelial cells and leukocyte infiltration as prognostic markers in colorectal cancer. Clin Gastroenterol Hepatol 4: 1351–1357 17059898 10.1016/j.cgh.2006.08.005

[bib3] Barrett KL, Willingham JM, Garvin AJ, Willingham MC (2001) Advances in cytochemical methods for detection of apoptosis. J Histochem Cytochem 49: 821–832 11410607 10.1177/002215540104900703

[bib4] Brown JM, Attardi LD (2005) The role of apoptosis in cancer development and treatment response. Nat Rev Cancer 5: 231–237 15738985 10.1038/nrc1560

[bib5] Cleven AH, van EM, Wouters BG, de Bruine AP (2007) Stromal expression of hypoxia regulated proteins is an adverse prognostic factor in colorectal carcinomas. Cell Oncol 29: 229–240 17452775 10.1155/2007/945802 PMC4617795

[bib6] de Bruin EC, Medema JP (2008) Apoptosis and non-apoptotic deaths in cancer development and treatment response. Cancer Treat Rev 34: 737–749 18722718 10.1016/j.ctrv.2008.07.001

[bib7] de Heer P, de Bruin EC, Klein-Kranenbarg E, Aalbers RI, Marijnen CA, Putter H, de Bont HJ, Nagelkerke JF, Van Krieken JH, Verspaget HW, van de Velde CJ, Kuppen PJ (2007) Caspase-3 activity predicts local recurrence in rectal cancer. Clin Cancer Res 13: 5810–5815 17908973 10.1158/1078-0432.CCR-07-0343

[bib8] de Oca J, Azuara D, Sanchez-Santos R, Navarro M, Capella G, Moreno V, Sola A, Hotter G, Biondo S, Osorio A, Marti-Rague J, Rafecas A (2008) Caspase-3 activity, response to chemotherapy and clinical outcome in patients with colon cancer. Int J Colorectal Dis 23: 21–27 17805550 10.1007/s00384-007-0362-3

[bib9] Hagg M, Biven K, Ueno T, Rydlander L, Bjorklund P, Wiman KG, Shoshan M, Linder S (2002) A novel high-through-put assay for screening of pro-apoptotic drugs. Invest New Drugs 20: 253–259 12201488 10.1023/a:1016249728664

[bib10] Hardwick JC, Kodach LL, Offerhaus GJ, van den Brink GR (2008) Bone morphogenetic protein signalling in colorectal cancer. Nat Rev Cancer 8: 806–812 18756288 10.1038/nrc2467

[bib11] Hilska M, Roberts PJ, Collan YU, Laine VJ, Kossi J, Hirsimaki P, Rahkonen O, Laato M (2007) Prognostic significance of matrix metalloproteinases-1, -2, -7 and -13 and tissue inhibitors of metalloproteinases-1, -2, -3 and -4 in colorectal cancer. Int J Cancer 121: 714–723 17455256 10.1002/ijc.22747

[bib12] Igney FH, Krammer PH (2002) Death and anti-death: tumour resistance to apoptosis. Nat Rev Cancer 2: 277–288 12001989 10.1038/nrc776

[bib13] Ishiguro K, Yoshida T, Yagishita H, Numata Y, Okayasu T (2006) Epithelial and stromal genetic instability contributes to genesis of colorectal adenomas. Gut 55: 695–702 16354798 10.1136/gut.2005.079459 PMC1856111

[bib14] Jonges LE, Nagelkerke JF, Ensink NG, van der Velde EA, Tollenaar RA, Fleuren GJ, van de Velde CJ, Morreau H, Kuppen PJ (2001) Caspase-3 activity as a prognostic factor in colorectal carcinoma. Lab Invest 81: 681–688 11351040 10.1038/labinvest.3780277

[bib15] Kaufmann SH, Lee SH, Meng XW, Loegering DA, Kottke TJ, Henzing AJ, Ruchaud S, Samejima K, Earnshaw WC (2008) Apoptosis-associated caspase activation assays. Methods 44: 262–272 18314058 10.1016/j.ymeth.2007.11.005

[bib16] Koornstra JJ, De Jong S, Hollema H, De Vries EG, Kleibeuker JH (2003) Changes in apoptosis during the development of colorectal cancer: a systematic review of the literature. Crit Rev Oncol Hematol 45: 37–53 12482571 10.1016/s1040-8428(01)00228-1

[bib17] Kumar S (2007) Caspase function in programmed cell death. Cell Death Differ 14: 32–43 17082813 10.1038/sj.cdd.4402060

[bib18] Langers AM, Sier CF, Hawinkels LJ, Kubben FJ, van DW, van der Reijden JJ, Lamers CB, Hommes DW, Verspaget HW (2008) MMP-2 geno-phenotype is prognostic for colorectal cancer survival, whereas MMP-9 is not. Br J Cancer 98: 1820–1823 18506186 10.1038/sj.bjc.6604380 PMC2410128

[bib19] Leers MP, Kolgen W, Bjorklund V, Bergman T, Tribbick G, Persson B, Bjorklund P, Ramaekers FC, Bjorklund B, Nap M, Jornvall H, Schutte B (1999) Immunocytochemical detection and mapping of a cytokeratin 18 neo-epitope exposed during early apoptosis. J Pathol 187: 567–572 10398123 10.1002/(SICI)1096-9896(199904)187:5<567::AID-PATH288>3.0.CO;2-J

[bib20] Leonardos L, Butler LM, Hewett PJ, Zalewski PD, Cowled PA (1999) The activity of caspase-3-like proteases is elevated during the development of colorectal carcinoma. Cancer Lett 143: 29–35 10465334 10.1016/s0304-3835(99)00176-7

[bib21] Lowry OH, Rosebrough NJ, Farr AL, Randall RJ (1951) Protein measurement with the Folin phenol reagent. J Biol Chem 193: 265–275 14907713

[bib22] Macheda ML, Stacker SA (2008) Importance of Wnt signaling in the tumor stroma microenvironment. Curr Cancer Drug Targets 8: 454–465 18781892 10.2174/156800908785699324

[bib23] Mesker WE, Junggeburt JM, Szuhai K, de Heer P, Morreau H, Tanke HJ, Tollenaar RA (2007) The carcinoma-stromal ratio of colon carcinoma is an independent factor for survival compared to lymph node status and tumor stage. Cell Oncol 29: 387–398 17726261 10.1155/2007/175276 PMC4617992

[bib24] Mueller MM, Fusenig NE (2004) Friends or foes – bipolar effects of the tumour stroma in cancer. Nat Rev Cancer 4: 839–849 15516957 10.1038/nrc1477

[bib25] Naber HP, ten DP, Pardali E (2008) Role of TGF-beta in the tumor stroma. Curr Cancer Drug Targets 8: 466–472 18781893 10.2174/156800908785699342

[bib26] Ngan CY, Yamamoto H, Seshimo I, Tsujino T, Man-i M, Ikeda JI, Konishi K, Takemasa I, Ikeda M, Sekimoto M, Matsuura N, Monden M (2007) Quantitative evaluation of vimentin expression in tumour stroma of colorectal cancer. Br J Cancer 96: 986–992 17325702 10.1038/sj.bjc.6603651 PMC2360104

[bib27] Porter AG, Janicke RU (1999) Emerging roles of caspase-3 in apoptosis. Cell Death Differ 6: 99–104 10200555 10.1038/sj.cdd.4400476

[bib28] Sonoshita M, Takaku K, Oshima M, Sugihara K, Taketo MM (2002) Cyclooxygenase-2 expression in fibroblasts and endothelial cells of intestinal polyps. Cancer Res 62: 6846–6849 12460897

[bib29] Stadelmann C, Lassmann H (2000) Detection of apoptosis in tissue sections. Cell Tissue Res 301: 19–31 10928278 10.1007/s004410000203

[bib30] Tsujino T, Seshimo I, Yamamoto H, Ngan CY, Ezumi K, Takemasa I, Ikeda M, Sekimoto M, Matsuura N, Monden M (2007) Stromal myofibroblasts predict disease recurrence for colorectal cancer. Clin Cancer Res 13: 2082–2090 17404090 10.1158/1078-0432.CCR-06-2191

