# Supplementary figures and images for: Clinical significance of stromal apoptosis in colorectal cancer
Source: Br J Cancer. 2009 Aug 4;101(5):765–73. doi: 10.1038/sj.bjc.6605220 (PMC2736838; doi:10.1038/sj.bjc.6605220)

## Slide 1
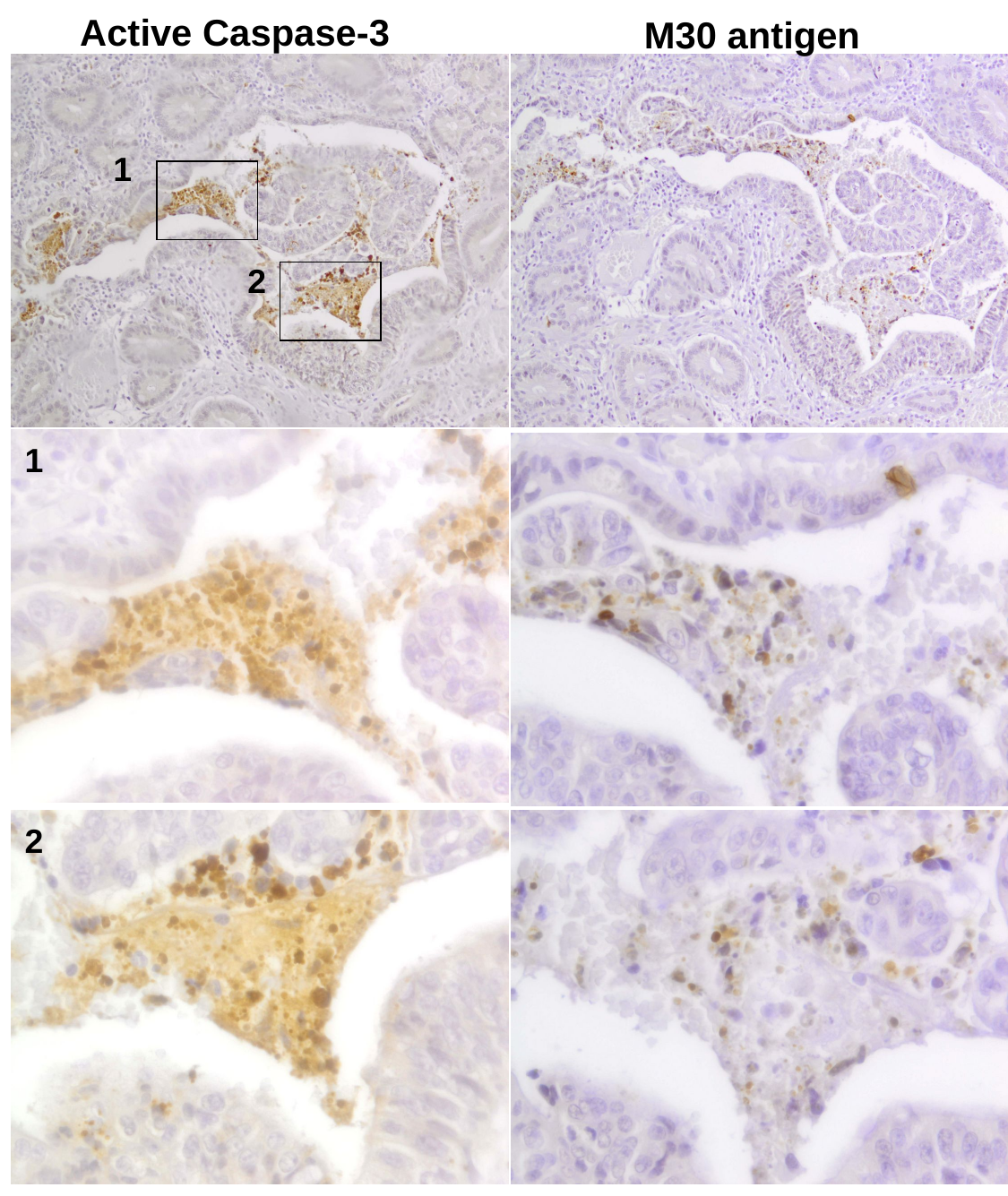

Active Caspase-3
M30 antigen
1
2
1
2

Supplement: Supplementary Figure [file 6605220x1.ppt]
