# Supplementary material for: Clinical significance of stromal apoptosis in colorectal cancer
Source: Br J Cancer. 2009 Aug 4;101(5):765–73. doi: 10.1038/sj.bjc.6605220 (PMC2736838; doi:10.1038/sj.bjc.6605220)
Supplement: Supplementary Figure Legend [file 6605220x2.doc]

**SUPPLEMENTARY FIGURE LEGENDS**

Supplementary figure 1*: Caspase-3 activity and M30 staining in CRC tumour 1.*

Some high power magnifications of tumour 1 from original Figure 1 to illustrate the diverse nature of stromal apoptosis are shown. Tumour with many cells expressing active caspase-3, not correlating with M30 staining.
